# Cervical spinal cord stimulation exerts anti-epileptic effects in a rat model of epileptic seizure through the suppression of CCL2-mediated cascades

**DOI:** 10.1038/s41598-024-64972-y

**Published:** 2024-06-24

**Authors:** Yosuke Okazaki, Tatsuya Sasaki, Kakeru Hosomoto, Shun Tanimoto, Koji Kawai, Takayuki Nagase, Chiaki Sugahara, Satoru Yabuno, Kyohei Kin, Susumu Sasada, Takao Yasuhara, Shota Tanaka, Isao Date

**Affiliations:** 1 Department of Neurological Surgery, Okayama University Graduate School of Medicine, Dentistry, and Pharmaceutical Sciences, 2-5-1 Shikata-cho, Kita-ku, Okayama 700-8558 Japan; 2 Department of Neurosurgery, Kure Kyosai Hospital, Kure, Japan; 3 Department of Neurosurgery, Okayama Rosai Hospital, Okayama, Japan

**Keywords:** Epileptic seizure, Glial cells, Spinal cord stimulation, C–C motif chemokine ligand 2, Diseases of the nervous system, Glial biology

## Abstract

Epidural spinal cord stimulation (SCS) is indicated for the treatment of intractable pain and is widely used in clinical practice. In previous basic research, the therapeutic effects of SCS have been demonstrated for epileptic seizure. However, the mechanism has not yet been elucidated. In this study, we investigated the therapeutic effect of SCS and the influence of epileptic seizure. First, SCS in the cervical spine was performed. The rats were divided into four groups: control group and treatment groups with SCS conducted at 2, 50, and 300 Hz frequency. Two days later, convulsions were induced by the intraperitoneal administration of kainic acid, followed by video monitoring to assess seizures. We also evaluated glial cells in the hippocampus by fluorescent immunostaining, electroencephalogram measurements, and inflammatory cytokines such as C–C motif chemokine ligand 2 (CCL2) by quantitative real-time polymerase chain reaction. Seizure frequency and the number of glial cells were significantly lower in the 300 Hz group than in the control group. SCS at 300 Hz decreased gene expression level of CCL2, which induces monocyte migration. SCS has anti-seizure effects by inhibiting CCL2-mediated cascades. The suppression of CCL2 and glial cells may be associated with the suppression of epileptic seizure.

## Introduction

Epilepsy is a common central nervous system disorder with a prevalence rate of approximately 1% of all humans^1^. The primary treatment for epilepsy is anti-seizure medications, which have been developed to target the neuronal cells. As mechanisms of epilepsy, the following three stages have been reported: (1) initial damage to the brain, such as febrile convulsions, head trauma, epileptic seizure, or stroke; (2) epileptogenic periods during which molecular or structural changes occur (the epileptogenesis was acquired in this period); and (3) chronic epilepsy characterized by the occurrence of spontaneous seizures^2,3^. It has been suggested that the acquisition of epileptogenesis in the second stage is responsible for subsequent seizure recurrence^2,3^. Clinically, it has been reported that it takes months to years to acquire the epileptogenesis^4,5^. On the other hand, in the basic research, it takes 10 to 30 days to acquire the epileptogenesis in the case of systemic administration of Kainic acid (KA)^6,7^. During this epileptogenic period, pathological evidence of neuronal loss in hippocampal CA1 and CA3^8^, sprouting of granule cells in the dentate gyrus, and the involvement of glial cells as well as neurons have been reported^9–11^. The mechanisms of this epilepsy have been also reported to include inflammation, cell death, and oxidative stress. Regarding the inflammation, the involvement of cytokine systems such as CCL2-CCR2 and the JAK2-STAT3 system has been reported. In this study, therefore, qRT-PCR testing was focused on the relationship between these molecules^12^^,^^13^.

Spinal cord stimulation (SCS) was known to the neuromodulation treatment in clinical practice for intractable neuropathic pain^14^, peripheral neuropathies^15^, angina pectoris^16^, and peripheral vascular disease^17^. The mechanism of the SCS was reported a decrease in glutamate concentration^18^, an increase in extracellular GABA concentration^19^, and inhibition of glial cell activation^20^. In the basic research, there are few relevant papers on the anti-seizure effects of SCS^13,21,22^. These papers reported that cortical epileptic waves are sensed and SCS stimulation may cause desynchronization, resulting in seizure suppression. However, the detailed molecular mechanisms have not yet been revealed. In this study, we therefore focused our attention on the glial cell interactions between epileptic seizures and SCS, and it is possible that SCS acts on the inflammatory mechanism of glial cells in epilepsy. Therefore, we investigated the effects of SCS therapy following KA-induced SE to reveal the molecular mechanisms and the involvement of glial cells.

## Material and methods

### Ethics statement and animal care

This study was conducted in accordance with the guidelines of the Institutional Animal Care and Use Committee of Okayama University Faculty of Medicine. The protocol was specifically approved by the Institutional Animal Care and Use Committee of Okayama University Faculty of Medicine (Protocol# OKU-2019651) and was conducted in accordance with the ARRIVE guidelines (https://arriveguidelines.org). Adult male Sprague–Dawley rats (Shimizu Laboratory Supplies Co., Ltd., Japan) weighing 250 to 300 g at the beginning of the study served as subjects for all experiments. Animal housing consisted of individual cages with one rat per one cage in a temperature- and humidity-controlled room that was maintained on a 12 h light/dark cycle with ad libitum access to food and water. Considering homeostatic control, all experiments were conducted during the daytime, and KA was administered between 8:00 A.M. and 11:00 A.M. Care was taken to record each animal at the same time of day. In addition, if the body weight decreased by more than 20% during the study, the experiment was terminated, and the animals were euthanized. In case of prolonged epileptic seizures longer than 30 min, diazepam (8 mg/kg) was administered intraperitoneally for its anti-seizure effect, in the interest of animal welfare.

#### Experiment 1 evaluation of a rat model of epileptic seizure by behavioral test, immunohistochemical investigation and electroencephalograms

The male Sprague–Dawley rats (n = 7 in each group) was received implantation of the device and spinal cord stimulation at day 0. Stimulation continued for 9 consecutive days^23^, with battery replacement every 3 days without generalized anesthesia. The animals were injected with KA (10 mg/kg) on day 2. The behaviors of rats were video monitored for 6 h after KA injection and seizure severity was evaluated by the Racine scale^24,25^. The animals were returned to the vivarium after the seizure evaluation period. Seven days after KA injection, rats underwent euthanasia and brains were harvested for immunohistochemical examination to evaluate glial cells: Ionized calcium binding adapter protein 1 (Iba-1), glial fibrillary acidic protein (GFAP) and 4′,6-diamidino-2-phenylindole (DAPI) staining). In another cohort, we also measured electroencephalograms (EEGs) for 6 h after the administration of KA (n = 3 in each group).

#### Experiment 2 evaluation of molecular mechanisms with qRT-PCR

**Figure 1:**
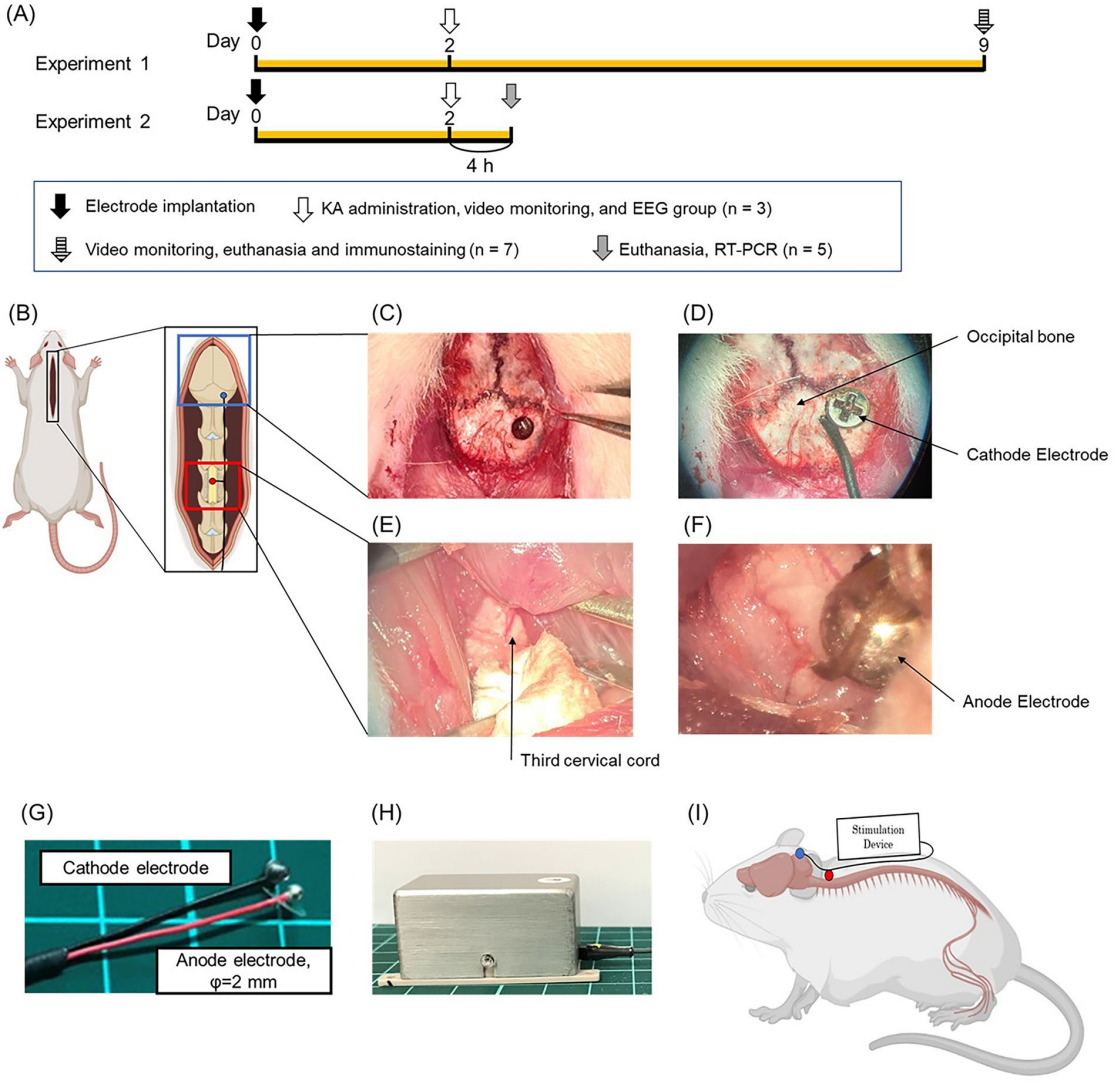
The images of SCS surgery. SCS electrode used in this study and experimental protocol (Experiment 1 and 2). (**A**) Experimental protocol of this study. Experiment 1 for control group, 2 Hz group, 50 Hz group, 300 Hz group (n = 7, respectively). In the stimulation group, the stimulation was initiated immediately after the surgery. Kainic acid was administered on day 2 and video monitoring was performed; the stimulation was continued for 1 week in the stimulation group after 6 h of recording, and video monitoring was done again on day 9 followed by sacrifice. Another group was also created in which kainic acid was administered on day 2 followed by EEG (n = 3). Experiment 2 for control group, 2 Hz group, 50 Hz group, 300 Hz group (n = 5, respectively). The rats were injected with kainic acid on day 2 to induce convulsions. They were sacrificed after 4 h for qRT-PCR. (**B**) An image showing the dorsal skin incision exposing the occipital bone and cervical spinal cord. (**C**–**F**) Images of a cathode and anode were implanted on the right occipital bone and C2 dura mater, respectively. (**G**, **H**) Images of the electrodes and stimulator used in this experiment. The stimulator is connected to the electrodes as shown in the figure. (**I**) The electrodes were led over the back skin and connected to the stimulator before being protected by a handmade jacket.

We performed qRT-PCR (n = 5 in control and 300 Hz group) to investigate the mechanism after confirming the anti-seizure effects for the high-frequency group (Fig. 1A). In the qRT-PCR group, the hippocampus was isolated from excised brains and evaluated for C–C motif chemokine ligand 2 (CCL2), C–C motif chemokine receptor 2 (CCR2), interleukin-6 (IL-6), IL-1β, tumor necrosis factor-α (TNF-α), Janus kinase 2 (JAK2), and signal transducer and activator of transcription 3 (STAT3), respectively.

### Surgical procedure of SCS therapy

SCS surgery was performed as described previously^26^. The rats were anesthetized with isoflurane and placed in a stereotaxic instrument (Narishige, Japan). The inhalation concentration of isoflurane was 5% at induction of anesthesia and then controlled at 1–3%. Following xylocaine injection to the midline of the head, animals underwent skin incision from the midline of the head to the back (Fig. 1B), and the spinal muscles were then carefully dissected so they were exposed and a C3 laminectomy was performed (Fig. 1E). We implanted a silver bipolar ball electrode (anode electrode) with a diameter of 2 mm epidurally on the dorsal surface of the spinal cord and fixed it to the muscle using 5–0 silk thread (Fig. 1F,G). We then drilled through the right occipital bone using a small hand drill and implanted a cathode electrode (grounding electrode) on the epidural space, with the lead tunneled subcutaneously to the back of the rats (Fig. 1C,D). Finally, the rats received a stimulation device that was fixed on their back using 1–0 silk thread at four fixing holes and encased in a protective jacket (Fig. 1H,I). The device is small, and the stimulation was performed continuously up to sacrifice. In the stimulation group, stimulation began immediately after surgery.

### Small mobile device for continuous electrical stimulation

We developed an electrical stimulation device called SAS-200 (Unique Medical Co., Ltd., Japan) that offered convenient adjustment of stimulation conditions via Bluetooth and allowed free movement of the rats owing to its small size^26,27^ (Fig. 1H). The SAS-200, which was attached to the back of the rats and was connected to the SCS electrode, delivered the stimulation. This stimulation required no anesthesia, thereby allowing rats to move around freely and making continuous stimulation possible. Additionally, the stimulation conditions could be easily adjusted wirelessly. The SAS-200 measured 20 mm × 40 mm × 20 mm, with a net weight of 26 g (including the battery). The stimulation parameters were as follows: pulse width, 100 µs; frequency, 2, 50, and 300 Hz; intensities (current stimulation), correspond to 80% of motor thresholds. It consisted of a control panel, a rechargeable lithium-ion battery, and an aluminum case^13^^,^^26^. For example, if the minimum value at which muscle contraction was seen with 1 mA stimulation, then the stimulation of that rat would be initiated at 0.8 mA. A standard Windows PC with a specific application controlled these stimulation conditions, namely, the beginning, duration, and particular conditions. A battery change involved simply removing the screws and replacing the depleted battery with a fully charged battery.

### Behavioral tests

We used the Racine scale (stage 1, absence-like immobility; stage 2, hunching with facial automatism and/or abducted forelimbs, wet-dog shaking; stage 3, rearing with facial automatism and forelimb clonus; stage 4, repeated rearing with continuous forelimb clonus and falling; and stage 5, generalized tonic–clonic convulsions with lateral recumbence or jumping) based on previous literature (Supplementary file 1)^24^. Video monitoring was performed for 6 h after the administration of KA, and the seizure frequency, the mean value of the Racine scale for each hour, and the time from administration of KA to convergence of seizures were evaluated. We always performed tests by the same, blinded researcher.

### Construction of electrode implantation and EEG recording

**Figure 2:**
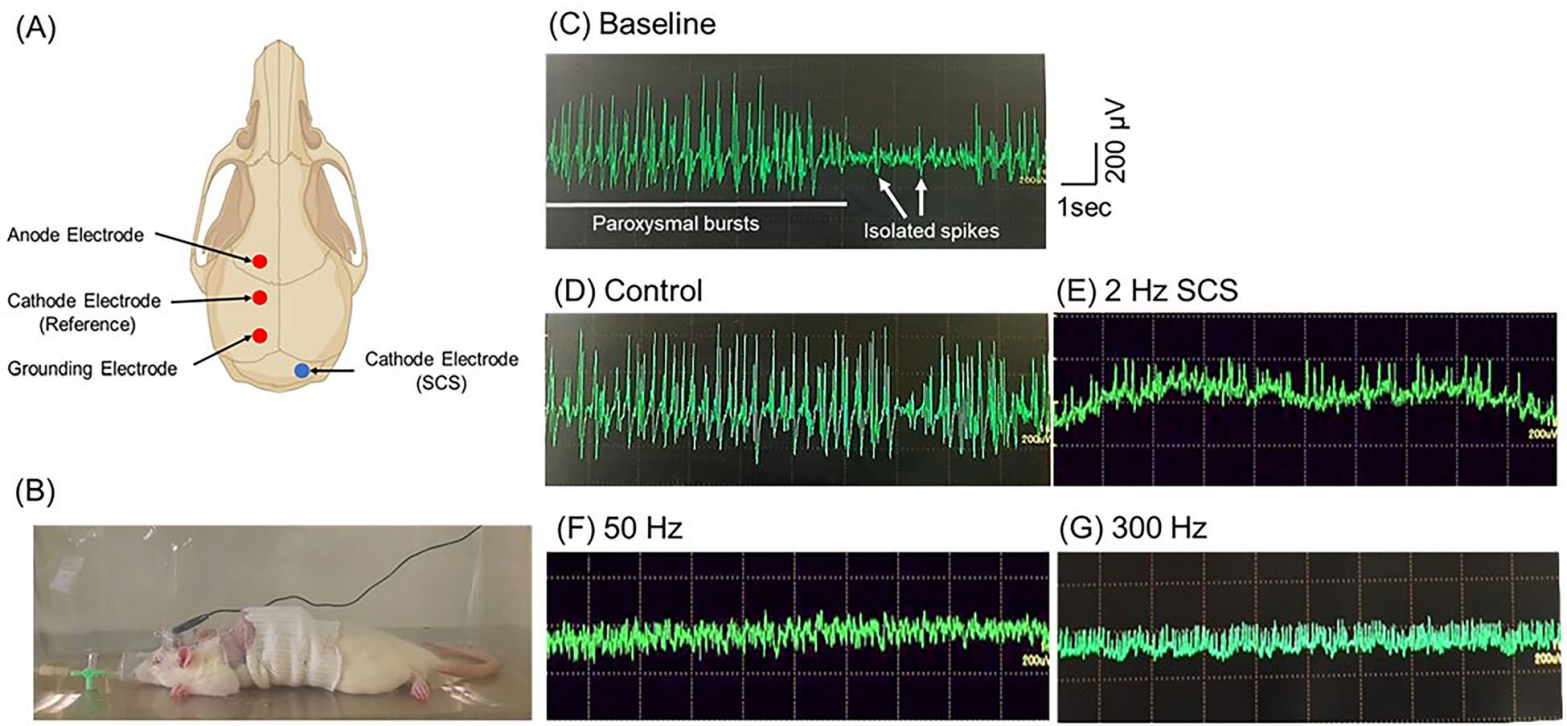
(**A**) The electrode configuration and examples of EEG recordings of KA model rat. (**B**) The image of rats during EEG recordings under general anesthesia. (**C**) The representative EEG recordings displaying activity during the acute phase of epilepsy. Two types of epileptiform events are distinguished: paroxysmal bursts and isolated spikes. (**D**–**H**) Representative EEG recordings for each group. Abbreviations: SCS, spinal cord stimulation; EEG, electroencephalogram.

After the rats were implanted with a ground electrode on the epidural space, we drilled a hole on the right frontal bone (anode) and two holes on the left parietal bone (reference and ground) (Fig. 2A). Next, animals received three epidural electrodes, which were fixed using dental cement. We secured a protective cap over the bone and the cord was housed inside. Two days after the operation, the animals were anesthetized with isoflurane again, and the EEG-electrodes were connected to the recording system (MEB-2200 Neuropack^®^, NIHON KOHDEN) and filtered (high-pass filter cut-off 0.5 Hz, low-pass filter cut-off 100 Hz). The animals were injected with KA solution (10 mg/kg), video EEG monitoring was performed for 6 h (Fig. 2B–G), and we evaluated the number of isolated spikes and paroxysmal bursts. The paroxysmal bursts was defined as deviation from other background activities and epileptiform discharge (spike or sharp wave) for more than 4 s in a sequence^28^.

### Immunohistochemical investigations and morphological analyses

Seven days after KA injection, each animal was anesthetized and perfused transcardially with 4% paraformaldehyde in 0.01 M phosphate-buffered saline (PBS). We then harvested the brains carefully, post-fixed them in the same fixative, and soaked them in 30% sucrose solution. The brains were coronally cut and then embedded in Tissue Tek O.C.T. compound (Sakura Finetek, Torrance, CA) and subsequently sectioned at 35 μm thickness using a Leica freezing microtome. The following primary antibodies were used for tissue staining: rabbit anti-Iba1 antibody (1:250; Wako Pure Chemical Industries, Osaka, Japan), rabbit anti-GFAP antibody (1:1000; Novus Biologicals, Littleton, CO). After rinsing in PBS, sections were incubated for 1 h in FITC-conjugated affinity-purified donkey anti-rabbit IgG (H + L) and 4,6-diamidino-2-phenylindole (DAPI; 2 drops/mL, R37606; Thermo Fisher, Waltham, MA) in a dark chamber. The sections were then extensively washed with PBS and coverslipped. The fluorescent immunoreactivities were visualized using an inverted fluorescence phase-contrast microscope BZ-X710 (Keyence, Osaka, Japan). The number of Iba-1 and GFAP cells in the CA1, CA3, and dentate gyrus was counted using six randomly selected regions (500 × 500 µm^2^, from 2.8 to 4.52 mm posterior from the bregma) in each rat.

### qRT-PCR

The hippocampus was collected immediately after decapitation 4 h after KA administration in the control group and the 300 Hz group (n = 5). Tissue samples were placed in individual tubes containing the tissue storage reagent and were stored at − 70 °C until RNA isolation. Syntheses of cDNA and qRT-PCR procedures were conducted as described previously^29,30^. As an internal control, we used GAPDH mRNA. The primer sequences used were as follows: CCL2: forward, GAG TAG GCT GGA GAG CTA CAA GAG; reverse, AGG TAG TGG ATG CAT TAG CTT CAG. CCR2: forward, CTT GTG GCC CTT ATT TTC CA; reverse, AGA TGA GCC TCA CAG CCC TA. IL-1β: forward, AGG CTT CCT TGT GCA AGT GT; reverse, TGA GTG ACA CTG CCT TCC TG. IL-6: forward, CCG GAG AGG AGA CTT CAC AG; reverse ACA GTG CAT CGC TGT TC. TNF-α: forward, CCA ACA AGG AGA AGT TCC; reverse, CTC TGC TTG GTG GTT TGC TAC. JAK2: forward, CTG AAA TCC TTG CAG CAT GA; reverse, CTC CAT GCC CTT GCA TAT CT. STAT): forward, CAG CCA AAC TCC CAG ATC AT; reverse, TCT GCT TTC ACA GCC ATC AC.

### Statistical analyses

We used GraphPad Prism ver.9.00 for Windows (GraphPad Software, San Diego, CA). Unpaired *t*-test or the Mann–Whitney *U* test was used for analyzing the qRT-PCR between the control group and the 300 Hz group. Single analysis of variance and the post hoc Tukey’s test were used for analyzing the number of Iba-1- and GFAP-positive cells in each seizure stage. Significant differences were preset as *p* < 0.05. Data are shown as means ± standard error (SE).

### Ethical publication statement

We confirm that we have read the Journal’s position on issues involved in ethical publication and affirm that this report is consistent with those guidelines.

## Results

### Body weight, stimulation frequency, and seizure frequency were not significantly different between the control and stimulation groups

**Figure 3:**
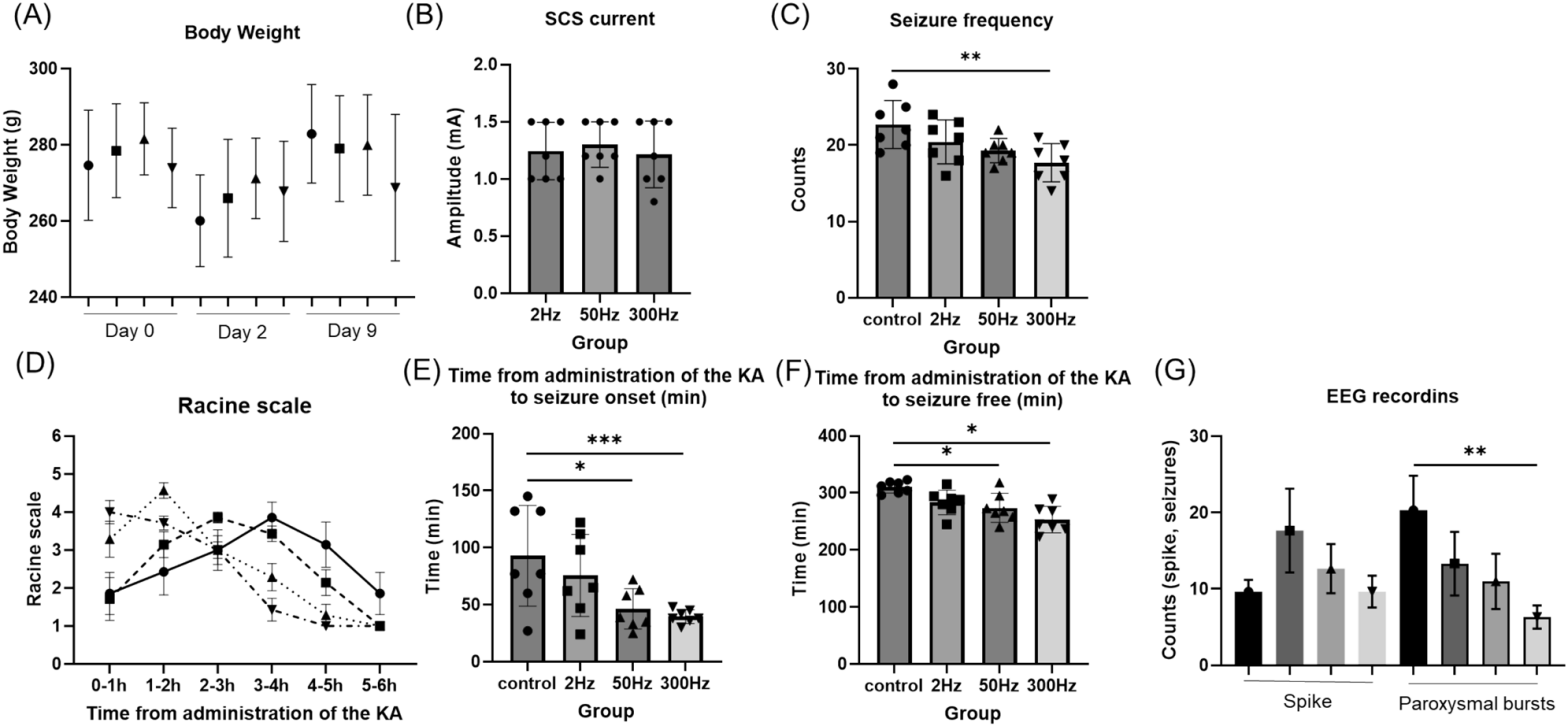
The results of body weight, SCS current, behavior test and EEG recordings. (**A**) In all groups, body weight decreased from day 0 to day 2, and increased from day 2 to day 9. Body weight was not significantly different in each group. (**B**) The current also did not differ significantly between groups. (**C**) The seizure frequency was significantly lower in the 300 Hz group than in the control group. (**D**) The Racine scale of the control group was gradually worse, with the worst results at 3–4 h. In the stimulation group, seizures were observed early and gradually disappeared. (**E**) The time from KA administration to seizure was significantly faster in the 50 Hz and 300 Hz groups than in the control group. (**F**) The time from KA administration to seizure free was also significantly faster in the 50 Hz and 300 Hz groups than in the control group. (**G**) Results of EEG recording: There was no difference in the number of isolated spikes, but the number of paroxysmal bursts was significantly lower in the 300 Hz group than in the control group. Data are presented as mean ± SE n = 7 rat for each group. * *p* < 0.05, ** *p* < 0.01, *** *p* < 0.001. Abbreviations: KA, kainic acid; EEG, electroencephalogram.

Body weights on day 0, 2, and 9 were not significantly different between the groups (*F*_(3,24)_ = 0.63, *p* = 0.602) (day 0: control group: 274.6 ± 14.45; 2 Hz group: 278.4 ± 12.33; 50 Hz group: 281.5 ± 9.48; 300 Hz group: 273.9 ± 10.46, respectively); (*F*_(3,24)_ = 0.913, *p* = 0.449) (day 2: control group: 260.1 ± 12.03; 2 Hz group: 266.0 ± 15.44; 50 Hz group: 271.2 ± 10.57; 300 Hz group: 267.8 ± 13.15, respectively); (*F*_(3,24)_ = 1.177, *p* = 0.339) (day 9: control group: 282.9 ± 12.94; 2 Hz group: 279.0 ± 13.87; 50 Hz group: 279.9 ± 13.17; 300 Hz group: 268.7 ± 19.23, respectively) (Fig. 3A).

The mean current at 2 Hz, 50 Hz, and 300 Hz for the stimulating groups was 1.24 ± 0.23, 1.30 ± 0.19, and 1.21 ± 0.27 (mA), respectively, and were not significantly different (*F*_(2,21)_ = 0.21, *p* = 0.810) (Fig. 3B).

The seizure frequency 6 h after KA administration was counted. The results for each group were 22.71 ± 2.91, 20.43 ± 2.66, 19.29 ± 1.48, and 17.71 ± 2.31 (count), respectively, significantly lower in the 300 Hz group than in the control group (*F*_(3,24)_ = 4.591, *p* = 0.011) (Fig. 3C).

### The time from KA administration to seizure onset and the time from KA administration to seizure suppression of the SCS stimulation group (50 Hz and 300 Hz) were shorter than those of control group

In terms of seizure severity, the control and stimulation groups had different tendencies. The Racine scale for severity of seizures showed that the more frequently stimulated group had more severe seizures earlier and that they converged more quickly. In contrast, in the control group, seizures gradually worsened, and had the worst outcome at 3 to 4 h (Fig. 3D). For each group, there were no obvious significant differences (*F*_(3,20)_ = 0.267, *p* = 0.8483). Specifically, the time from KA administration to seizure onset was 92.86 ± 40.89, 75.71 ± 33.29, 46.43 ± 16.42, 39.57 ± 5.55 (min), and the time from KA administration to seizure suppression was 310.14 ± 9.52, 283.43 ± 19.97, 273.86 ± 23.62, and 253.29 ± 21.49 (min). Significant differences were found among the 50 Hz, 300 Hz, and control groups in both analyses (time from KA administration to seizure onset:* F*_(3,24)_ = 4.869, *p* = 0.0087; time from KA administration to seizure suppression: *F*_(3,24)_ = 8.859, *p* = 0.0004) (Fig. 3E,F).

### The 300 Hz group had significantly less counts of paroxysmal bursts than the control group in the electroencepharoglam

The number of isolated spikes was 19.67 ± 1.25, 17.67 ± 4.50, 12.67 ± 2.62, and 10.0 ± 2.45 (/6 h) in the control, 2 Hz, 50 Hz, and 300 Hz groups, respectively, with no apparent significant difference (*F*_(3,8)_ = 3.613, *p* = 0.065). The number of paroxysmal bursts was 20.33 ± 3.68, 13.33 ± 3.40, 11.0 ± 2.94, and 6.33 ± 1.25 (/6 h) in each group, respectively, and was significantly less in the 300 Hz group than in the control group (*F*_(3,8)_ = 7.704, *p* = 0.0096) (Fig. 3G). In addition, there were more paroxysmal bursts than spikes in the control group, but more spikes than paroxysmal bursts in all stimulation groups.

### In the 300 Hz group, both Iba-1-positive and GFAP-positive cells were significantly reduced

**Figure 4:**
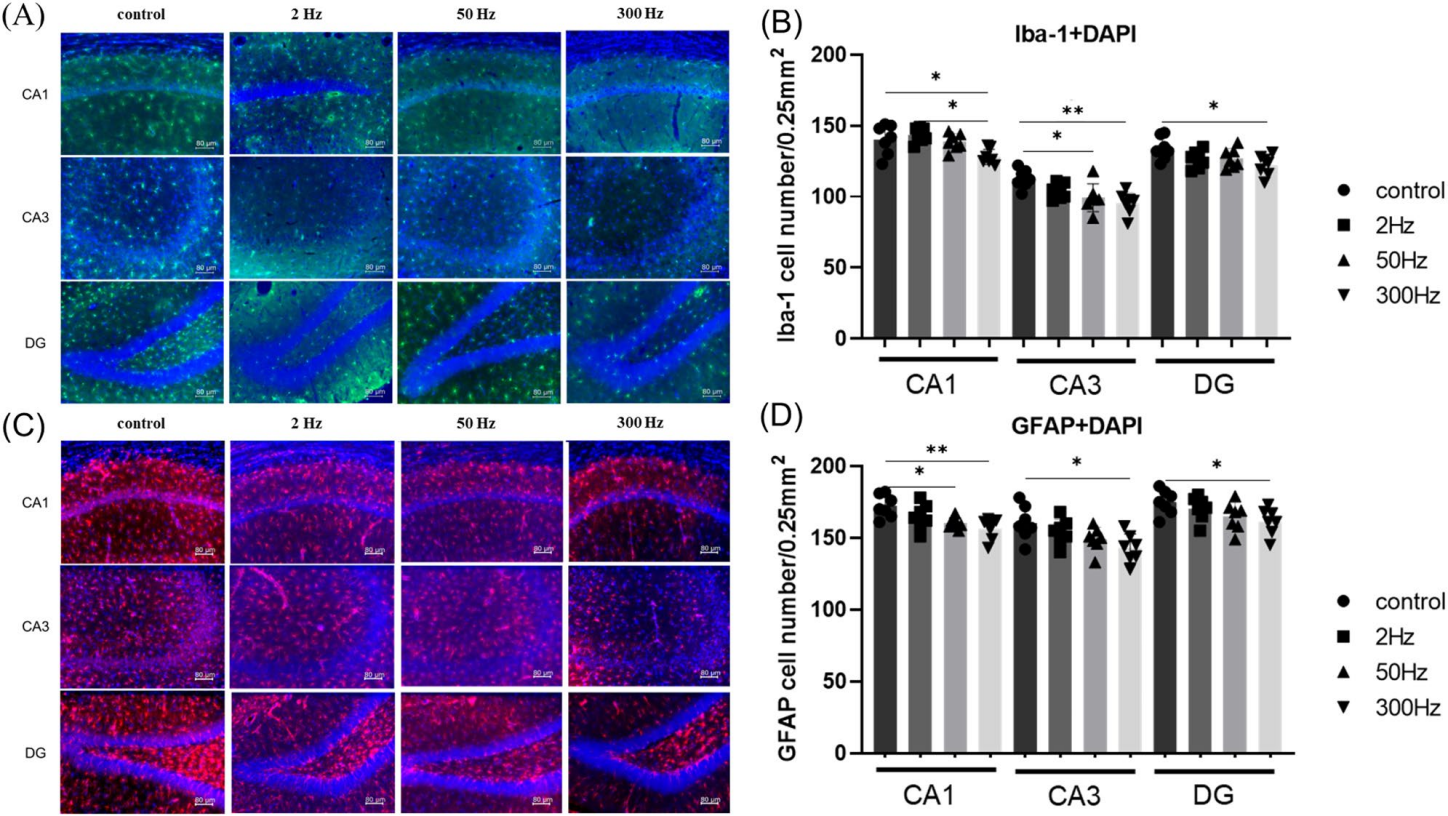
(**A**, **C**) Double immunofluorescence staining of DAPI (blue) with Iba-1 (green) and GFAP (red) in the hippocampal CA1, CA3, and DG 7 days after the administration of KA. Scale bar = 80 µm. (**B**, **D**) Quantitative data of d showing the number of Iba1- and GFAP-positive cells in the hippocampus. Data are presented as the mean ± standard error. n = 7 rats for each group. *p < 0.05, **p < 0.01, ***p < 0.001. Abbreviations: Iba-1, ionized calcium-binding adapter molecule 1; GFAP, glial fibrillary acidic protein; DAPI, 4,6-diamidino-2-phenylindole; DG, dentate gyrus; KA, kainic acid.

The number of Iba-1-positive cells was significantly fewer in the 300 Hz group than in the control group for CA1, CA3, and DG regions, and the 50 Hz group also showed significantly fewer Iba-1-positive cells than the control group in the CA3 region (CA1: *F*_(3,24)_ = 5.947, *p* = 0.0035; CA3: *F*_(3,24)_ = 6.696, *p* = 0.0019; DG: *F*_(3,24)_ = 3.362, *p* = 0.0353) (Fig. 4A,B). The number of GFAP-positive cells in the 300 Hz group was significantly fewer than that of the control group in the CA1, CA3, and DG regions, and the 50 Hz group also showed significantly fewer GFAP-positive cells than the control group in the CA1 region (CA1: *F*_(3,24)_ = 6.140, *p* = 0.0030; CA3: *F*_(3,24)_ = 4.010, *p* = 0.0191; DG: *F*_(3,24)_ = 2.987, *p* = 0.0511) (Fig. 4C,D).

### The 300 Hz group showed a reduction in CCL2 and TNF-α, which are involved in the inflammatory cascade, whereas IL-1β was increased

**Figure 5:**
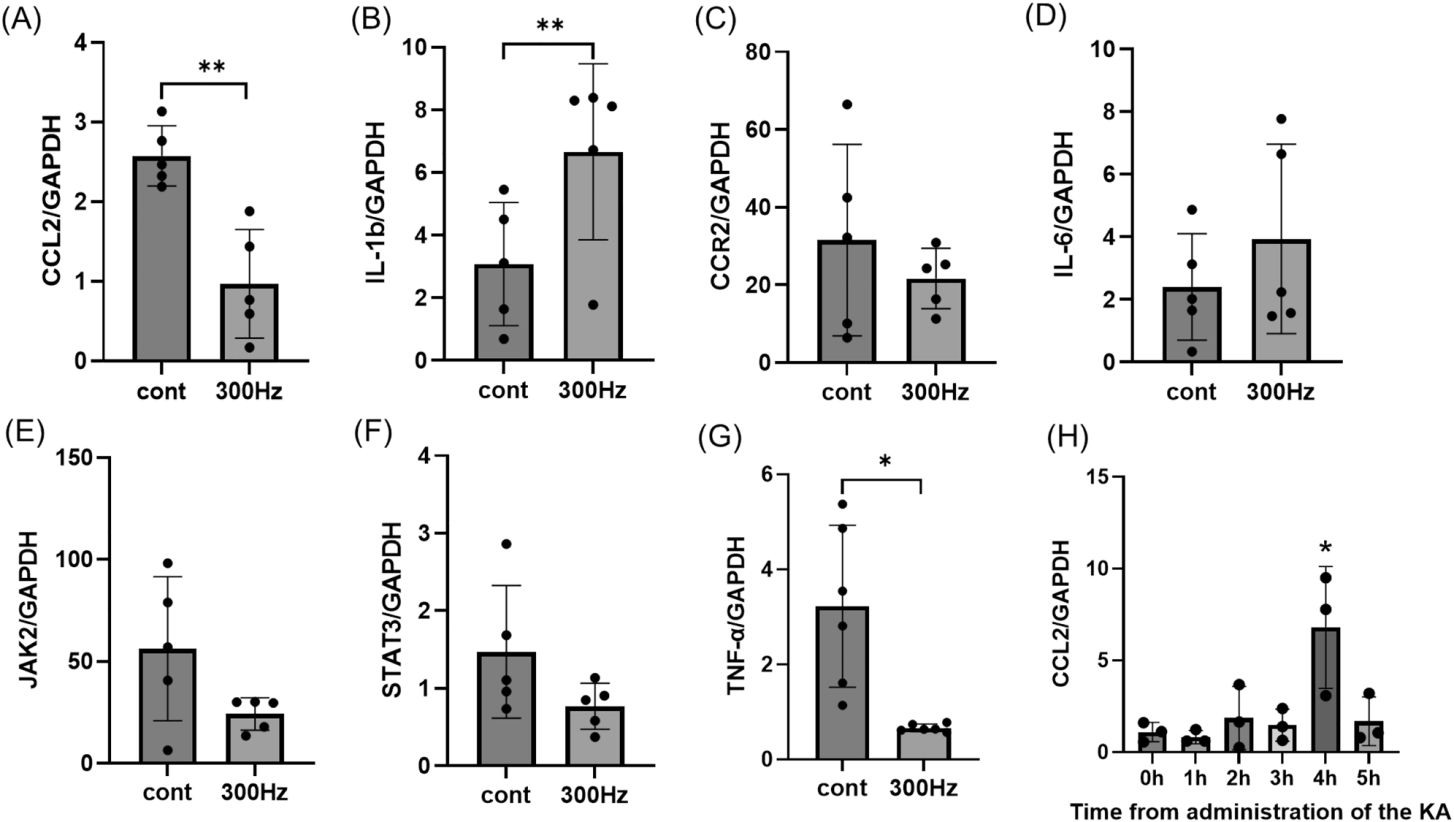
CCL2 and other inflammatory genes are shown in the hippocampus 4 h after administration of KA. (**A**, **G**) The expression of CCL2 and TNF-a was significantly decreased in the 300 Hz group compared to the control group. (**B**) The expression of IL-1β was also lower in the control group than in the 300 Hz group. (**C**–**F**) There were no differences in the expression of CCR2, JAK2, STAT3, and IL-6 between the two groups. (**H**) The expression of CCL2 was examined every hour after KA administration and was highest at 4 h. Data are presented as the mean ± standard error. n = 5 rats for each group. *p < 0.05, **p < 0.01, ***p < 0.001. Abbreviations: CCL2, C–C motif chemokine ligand 2; IL-1β, interleukin-1β; CCR2, C–C motif chemokine receptor 2; IL-6, interleukin-6; JAK2, Janus kinase 2; STAT3, signal transducer and activator of transcription 3; TNF-α, tumor necrosis factor-α; KA, kainic acid.

The CCL2 expression in the control group after KA administration peaked at 4 h (Fig. 5H). Therefore, we decided to perform qRT-PCR in the control and 300 Hz groups 4 h after KA administration. The CCL2 and TNF-α mRNA levels were downregulated in the 300 Hz group compared with the control group (CCL2: *p* = 0.0018, TNF-α: *p* = 0.0043), but the expression of IL-1β was significantly decreased in the control group (*p* = 0.048) (Fig. 5A,B,G). The expression of CCR2, JAK2, STAT3, and IL-6 between the two groups was not significantly difference (Fig. 5C–F).

## Discussion

In the present study, we were able to show the antiepileptic effects of SCS in stimulation group using both behavioral and electrophysiological evaluations. The mechanisms were suggested to involve glial cell inhibition and CCL2-mediated networks, both of which are assumed to be closely related to the inflammatory cascade. Jiao et al. evaluated the anti-seizure effects of SCS in a pentylenetetrazol-induced seizure rat model^13^. They used four stimulus groups: 30 Hz, 80 Hz, 130 Hz, and 180 Hz. The results showed that both the current and frequency of seizure waves were improved in the high-frequency stimulation groups of 130 Hz and 180 Hz. They proposed a mechanism in which SCS stimulates the right somatosensory hindlimb area in the dorsal columns, which is the pathway that leads to the thalamus via the somatosensory tract, so stimulation of the dorsal spinal cord by SCS is an indirect method of stimulating the thalamic nucleus and may therefore induce effects like those of thalamic deep brain stimulation^31,32^. Other reported mechanisms of action for SCS include the increased extracellular GABA concentration in the spinal cord dorsal horn, decreased glutamate concentration, suppressed glial cell activation, and decreased GFAP and CCL2^18,20^, similar to our results.

Sano et al. reported that microglia are activated in the early stages of epileptogenesis and release TNF-α and IL-1β, which in turn activate astrocytes. They also suggested that activated microglia bind to P2Y12 receptors and exhibit neuroprotective effects, while producing inflammatory cytokines such as IL-1β, TNF-α, and IL-6^33^. Tian et al.^12^ reported that the release of CCL2 by KA administration results in the secretion of cytokines such as TNF-α and the release of IL-1β by inducing the JAK2-STAT3 pathway. Although there was no difference in these JAK2-STAT3 pathways in the present study, we only took measurements at 4 h post-seizure this time and the changes may be observed along the time course. In addition, Stephens et al. reported that the most significant changes in genetic analysis reported by SCS were related to immune response and synaptic signaling^34^. From these reports, we hypothesized that suppression of CCL2 after KA administration may inhibit the activation of microglia and astrocytes, and consequently contribute to the release of inflammatory cytokines and suppression of seizures.

**Figure 6:**
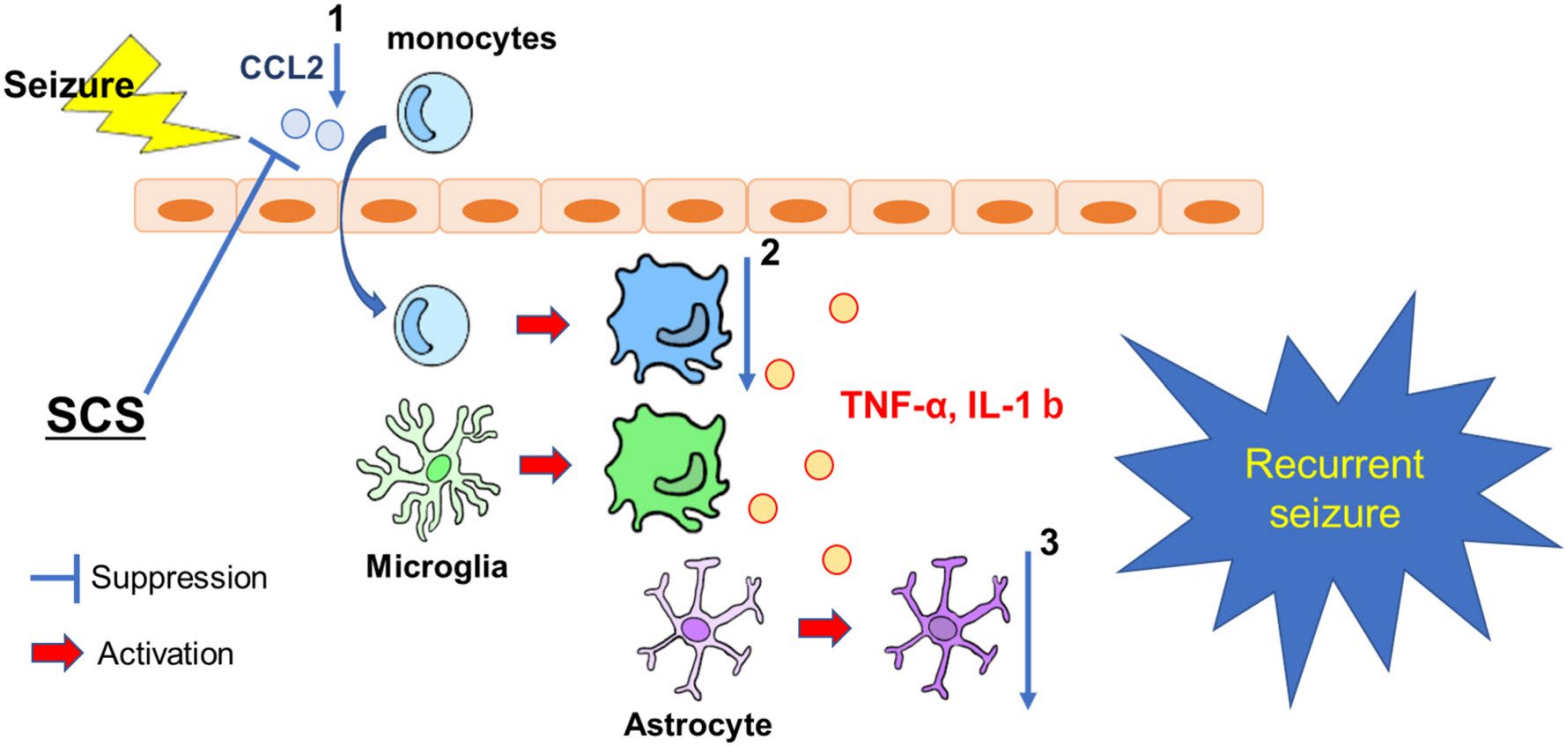
Seizures induce an upregulation of CCL2 expression. This causes monocytes in blood vessels to be transported into the brain, resulting in activation. Microglia in the brain are also activated by seizures, and both release inflammatory cytokines such as TNF-α and IL-1b, which in turn activate astrocytes. From the present study, the suppression of CCL2 in the high-frequency stimulation group may reduce the number of monocytes transported into the brain (1), which may have contributed to the suppression of inflammatory cytokines (2), the suppression of activation of astrocytes (3) and the reduction of seizures. Abbreviations: CCL2, C–C motif chemokine ligand 2; TNF-α, tumor necrosis factor-α; IL-1β, interleukin-1β; SCS, spinal cord stimulation.

The monocytes transported into the brain by CCL2 are activated and differentiated into macrophages. They then function in the same role as the microglia that are endogenous to the brain^35^. Tian et al.^12^ suggested that infiltrate monocytes could contribute to pathological progression of epilepsy on the CCL2-CCR2 chemokine signaling pathway and reduced infiltration in CCR2 knockout mice. In the present study, we showed that CCL2 and TNF-α expression was decreased in the 300 Hz group, and this was suggested that migrated monocytes could express less, resulting in a reduced inflammatory response and fewer epileptic seizures in the 300 Hz group (Fig. 6). To distinguish between infiltrated monocytes and resident microglia, it may be useful to evaluate, for example, P2Y12 staining, which stains microglia rather than monocytes^36^. Past findings also suggested that CCL2 is expressed by multiple cell types in the central nervous system, including neurons, microglia, endothelial cells, and astrocytes^37,38^. In the present study, it is not clear which cells released CCL2 and how it was released during epileptic seizures. Therefore, it would be meaningful to investigate this issue in the future.

In this experiment, IL-1β was elevated in the stimulation group unexpectedly. Bosco et al. reported the suppression of monocyte increase and suppression of IL-1β expression after seizures in a CCL2-CCR2 pathway–regulated study using a CCR2 knockout mouse model^35^. Regarding changes in IL-1β and other factors after seizures, Vezzani et al. reported that IL-1β in the hippocampus peaks 6 h after seizure induction and is followed by an increase in IL-1RA, an antagonist of IL-1β^39^. Clinical studies have reported that IL-1β is expressed in lesions of cortical dysplasia in patients with epilepsy and that the number of IL-1β-positive cells correlates with seizure frequency, thus the expression of IL-1β is an important factor in epileptogenesis^40^. Compared to their results, ours showed no suppression of IL-1β in the high-frequency group and severe seizures were observed earlier in the high-frequency stimulation group. There have also been reports of increased blood flow to cerebral infarction and cerebral vasospasm after subarachnoid hemorrhage due to SCS^41,42^. Therefore, we thought that SCS-induced vasodilatation and increased cerebral blood flow resulted in early and severe seizures with increased IL-1β expression^43^. Another reason might be that in the stimulation group, the slightly longer operative time due to the more careful electrode placement may have prolonged the postoperative inflammation.

## Conclusions

In the present study, high-frequency SCS showed a significant therapeutic effect on a rat model of SE, which is consistent with the results of previous reports. We also believe that high-frequency SCS contributed to this anti-seizure effect by regulating monocyte migration through the suppression of CCL2, thereby inhibiting the activation of glial cells in the brain. Although the clinical practice for epilepsy patients and the basic research for KA-induced epileptic seizure model of rats is different, the results suggest that SCS may help to reveal the mechanism of status and contribute to the treatment of epilepsy in the future.

### Supplementary Information

Supplementary Legends.

Supplementary Figure S1.

## Data Availability

All data supporting the findings of this study are available within the paper.
