# Supplementary material for: Cervical spinal cord stimulation exerts anti-epileptic effects in a rat model of epileptic seizure through the suppression of CCL2-mediated cascades
Source: Sci Rep. 2024 Jun 24;14:14543. doi: 10.1038/s41598-024-64972-y (PMC11196670; doi:10.1038/s41598-024-64972-y)
Supplement: Supplementary file 1 — Supplementary Legends. [file 41598_2024_64972_MOESM1_ESM.docx]

Supplementary material

Figure S1 The schema of the seizure as the Racine scale. It showed; stage 1, absence-like immobility; stage 2, hunching with facial automatism and/or abducted forelimbs, wet-dog shaking; stage 3, rearing with facial automatism and forelimb clonus; stage 4, repeated rearing with continuous forelimb clonus and falling; and stage 5, generalized tonic–clonic convulsions with lateral recumbence or jumping.
