# Supplementary material for: Cervical spinal cord stimulation exerts anti-epileptic effects in a rat model of epileptic seizure through the suppression of CCL2-mediated cascades
Source: Sci Rep. 2024 Jun 24;14:14543. doi: 10.1038/s41598-024-64972-y (PMC11196670; doi:10.1038/s41598-024-64972-y)
Supplement: Supplementary file 2 — Supplementary Figure S1. [file 41598_2024_64972_MOESM2_ESM.pdf]

Supplementary file 1

Score	Exibited behavior
1	Normal behavior, absence-like immobility
2	Hunching with facial automatism and/or abducted forelimbs, wet-dog shaking
3	Rearing with facial automatism and forelimb clonus
4	Repeated rearing with continuous forelimb clonus and falling
5	Generalized tonic–clonic convulsions with lateral recumbence or jumping
